# Comparison of two different barbed suture materials for end-to-end jejuno-jejunal anastomosis in pigs

**DOI:** 10.1186/s13028-018-0437-x

**Published:** 2019-01-05

**Authors:** Gessica Giusto, Selina Iussich, Massimiliano Tursi, Giovanni Perona, Marco Gandini

**Affiliations:** Department of Veterinary Sciences, University of Turin, Largo P. Braccini 2-5, Grugliasco, TO Italy

**Keywords:** Barbed suture, End-to-end anastomosis, Jejunojejunal, Pigs

## Abstract

**Background:**

Hand-sewn intestinal anastomoses are a fundamental procedure in both open and laparoscopic intestinal surgery. Self-retaining barbed suture devices have been tested for a variety of surgical applications. With the exception of clinical reports and various experimental studies on enterotomy, little has been published so far on the use of barbed suture for end-to-end intestinal anastomoses. The aim of the study was to compare two different barbed suture materials for end-to-end jejuno-jejunal anastomosis in pigs. End-to-end jejuno-jejunal anastomosis were performed with unidirectional barbed (A group), bidirectional barbed (B group) or normal (C group) sutures in each animal. A comparison was then made between the groups based on adhesions scoring, suturing time, bursting pressure and histopathology.

**Results:**

Mean construction times in the A group (518 ± 40 s) and in the B group (487 ± 45 s) were significantly lower than in the C group (587 ± 63 s) but were not different between A and B group (P = 0.10). Mean bursting pressures were significantly higher in the intact intestine (197 ± 13 mmHg) than in any other group (group A 150 ± 16 mmHg, group B 145 ± 22 mmHg, group C 145 ± 24 mmHg). Among anastomotic techniques, the bursting pressures were not significantly different. Histologically no difference could be detected in the grade of inflammation, collagen deposition and neovascularization at the anastomotic sites.

**Conclusions:**

Barbed sutures can be effectively used for handsewn end-to-end jejunojejunal anastomosis in pigs. They are comparable to normal suture but could provide a shorter surgical time.

## Background

Despite the introduction of mechanical staplers, the importance of hand-sewn intestinal anastomoses remains uncontested in abdominal surgery, in both open and laparoscopic procedures. Self-retaining (i.e. barbed) suture devices have recently come under focus for a variety of surgical applications, including plastic, orthopedic, abdominal and urologic surgery [1–3]. Although still considered off-label, this newer material has already been employed in gastrointestinal surgery in both humans and animals [4–12]. With the exception of clinical reports mostly on side-to-side anastomosis [8] and various experimental studies on enterotomy [5, 9–11], little has been published so far. The most interesting application of barbed sutures is in plastic and laparoscopic surgery because of their handling characteristics but their use has been described in in-vivo open surgery techniques in humans [9, 13–17] and animals [12, 18–24]. Barbed sutures have proven effective in performing end-to-end anastomosis ex-vivo in humans [9], dogs [25], and horses [4]; however, no experimental study has evaluated the characteristics of jejuno-jejunal anastomoses in vivo to date.

Barbed sutures incorporate tiny barbs cut into the body of the filament, so that tissues can be approximated without the need for knots. Although barbed suture materials have been evaluated in clinical experience with positive results [5, 6, 8], concerns remain over the higher risk of inflammation and/or adhesion formation [26, 27] and increased susceptibility to complications [27–30] especially with the use of a unidirectional barbed suture [30]. These complications arise mostly from barbs that remain exposed at the suture end that can cause damage to organs in the surgical field [27–30] despite having been cut flush to the surface of the tissue [27]. One may hypothesize that in an intestinal end-to-end anastomosis a purse-string effect could be produced as a result of intestinal contractions pushing the intestinal wall along the suture while the barbed suture concurrently prevents return to its natural, designated position.

Furthermore, except for gastropexy in dogs [31], only the unidirectional glycomer-based barbed suture has been described for gastrointestinal applications; differences in conformation [32], handling, and postoperative complications exist between unidirectional and bidirectional barbed sutures [30] that warrant further evaluation of the application of bidirectional barbed suture in gastrointestinal surgery. In a recent review performed in human patients [30], the use of a unidirectional barbed suture resulted in reduced operative time but increased complications compared to the use of conventional sutures; the bidirectional barbed suture was comparable to conventional sutures regarding operative time and complications, although differences do exist in different types of surgery [30].

The aim of this study was to compare two different types of barbed sutures with smooth suture material for end-to-end, jejuno-jejunal anastomosis in pigs, with reference to the following: (a) surgery time, (b) complications, (c) adhesion formation, (d) bursting pressure, and (e) tissue healing.

We hypothesized that bidirectional barbed sutures would be faster to use, but would withstand the same bursting pressure and would not cause more complications than their unidirectional counterparts for end-to-end jejuno-jejunostomies. We also postulated that no migration of the intestinal wall along the suture would occur, and therefore no purse-string effect would develop.

## Methods

The study protocol was approved by the Bioethical Committee of the University of Turin and by the Italian Ministry of Health. A sample size calculation was performed using a freely available online sample size calculator (http://www.openepi.com), with alpha level of 0.05 and 80% power, based on bursting pressure. We used six large white/landrace cross-breed female pigs weighing 35 ± 5 kg. Animals were fasted for 12 h before surgery, but had free access to water. All pigs were sedated with xylazine^1^ (2 mg/kg, intramuscularly [IM]) and anaesthesia was induced with tiletamine and zolazepam^2^ (4.4 mg/kg, IM) and maintained with isoflurane (see footnote 2) in oxygen under spontaneous ventilation. Animals were placed in dorsal recumbency and the abdomen was surgically prepared. A laparotomy was performed through the *linea alba* to expose the small intestine. Starting 30 cm distally to the suspensory ligament of the duodenum, six resections were performed on the jejunum approximately 40 cm apart from each other. Intestinal continuity was restored with a jejuno-jejunal, end-to-end anastomosis in a continuous, appositional, extra-mucosal pattern [33–35]. Six anastomoses were created in each animal as follows: two using USP 4-0, unidirectional, barbed polyglycomer 631^3^ and a 26 mm, half-circle, taper-point needle (group A); two using USP 3-0, bidirectional, barbed polydioxanone^4^ and a 26 mm half-circle taper-point, double needle (group B); and two using USP 4-0, plain glycomer 631 (see footnote 3) and a 26 mm half-circle taper-point needle (group C). Suture materials were employed in a randomly assigned order, using a random number generator (http://www.random.org).

To provide consistency, all anastomoses were performed by the same surgeon (MG) after having undergone training in the use of barbed sutures in end-to-end anastomoses ex-vivo. Animals were treated preoperatively with a single administration of benzylpenicillin–dihydrostreptomycin^5^ (20 mg/kg, intramuscularly), while post-operative analgesic therapy consisted of intramuscular buprenorphine^6^ (0.01 mg/kg SID) for 72 h post-surgery. During recovery, pigs were placed under an infrared heat lamp. After recovery, access to water and food was allowed after 6 and 18 h, respectively.

### Surgical techniques

**Fig. 1:**
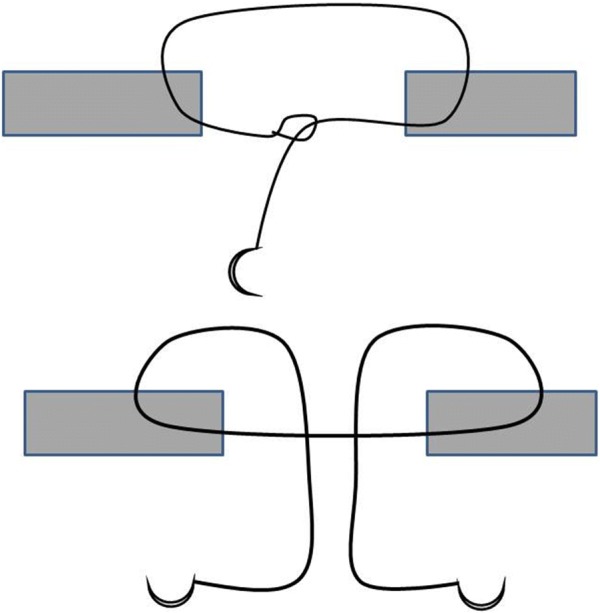
Diagram demonstrating step by step procedure for the continuous modified extramucosal pattern used in the present study

The intestine was severed transversely with a 60° inclination on both intestinal ends to avoid a stenotic anastomosis. The resulting wedge of tissue between the two ends was excised. Two plain glycomer 631 (see footnote 3) stay sutures were placed on the mesenteric and antimesenteric sides. Sutures were not tied; instead, their ends were held with mosquito forceps by an assistant surgeon. Stay sutures were removed after completion of the procedure. Anastomoses were sealed in a continuous, appositional, extramucosal pattern, which was modified according to the order of bites into the tissue (Fig. 1). The suture was placed so as to initially bury the knot (or the initial loop) into the submucosa and advanced with partial thickness bites, placed in a diagonal direction (while transverse passages were placed extraluminally to approximate edges). The suture pattern was initiated differently to suit the type of material used; however, the pattern itself was identical in all cases. Differences in initiating the pattern are detailed below.

#### Barbed polyglycomer 631 (group A)

This suture material is supplied with a welded loop at the end opposite to the needle and has unidirectional barbs cut along its length. The first bite, started from one cut edge of the intestine and catching the submucosa, exited from the serosa before again entering from the serosa and exiting from the other cut edge of the intestine, before feeding the needle back into the loop (Fig. 1). The suture was run for 180° in a continuous, appositional, extramucosal pattern, interrupted by an overlapping loop (made by backing over the suture) as previously described [4] and then continued for the remaining 180°. To secure the end of the suture line, two additional bites were taken once the anastomosis was completed. The first bite overlapped the beginning of the suture line, while the second backed over in the opposite direction. Finally, the suture was cut flush with the surface of the intestine.

#### Barbed polydioxanone (group B)

This suture material is supplied with two needles, using one at each end. The filament is divided into two half-portions with barbs arrayed in opposite directions (bidirectional) from the midpoint. To create an anastomosis, we began by placing two stay sutures, one on the mesenteric and one on the antimesenteric side. Then one needle was inserted in an extramucosal pattern from the cut edge of the intestine on each jejunal stump without completely pulling the suture out, but leading to the formation of a loop. Then, both needles were fed into the loop thus formed at the middle of the suture (Fig. 1). At this point, each side of the anastomosis was sealed in a continuous, modified, extramucosal pattern, using one needle for each side. As above, two additional bites were taken to lock the suture in place at the point the half-circumference was completed.

#### Unbarbed glycomer (group C)

After placing the two stay sutures, the anastomosis was completed in a modified, continuous, appositional extramucosal pattern, starting on the mesenteric side and burying the initial knot submucosally. The suture was tied at the antimesenteric side and continued until completion of the circumference.

The abdomen was lavaged with warm Ringer’s solution and closed in two layers.

On postoperative day 7, animals were again anaesthetized as described above and euthanized by intracardiac injection of embutramide, mebenzonium iodide, and tetracaine hydrochloride solution.^7^ Necropsy was performed by an operator who was blinded to the suture materials used. The following necropsy findings were recorded: (a) adhesions at the site, and distant from the site, of anastomosis; (b) intestinal stenosis (defined as the presence of a dilated portion of the intestine proximal to the anastomosis [36]; (c) leakage (defined as the leaking of intestinal content at the anastomotic sites after gentle pressure is applied proximally); and (d) presence of abscesses or granulomas at the anastomotic sites.

**Fig. 2:**
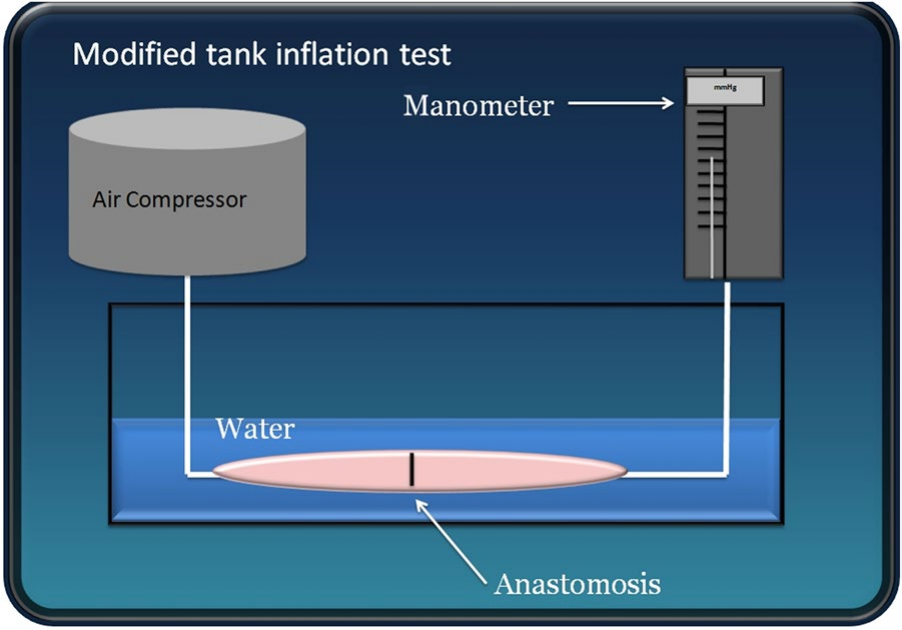
Diagram demonstrating the system used for bursting pressure measurements of the anastomoses

Adhesions were scored using the method implemented by Demyttenaere [5] (Table 1). Those that could be separated by applying gentle traction were released. Bursting pressure of the anastomosis was measured using an inflation tank test as previously described [37] (Fig. 2). Briefly, the intestine was severed 10 cm proximally and 10 cm distally to the anastomotic site. Next, the two ends were closed with plastic tie-wraps. A 20 G needle attached to a column manometer was tunneled through the intestinal wall at one end. At the opposite end, another 20 G needle attached to an air compressor was inserted in the same fashion. The entire specimen was held underwater as the air compressor began inflating at a rate of 0.5 L/min. The entire procedure was digitally filmed. Anastomotic leakage and bursting were indicated by air bubbles in the water tank and by a sudden pressure drop as measured by the manometer. The exact peak pressure was reported with the help of videography. The bursting pressures of 12 intact intestinal samples harvested from the same animals were also recorded as controls.

**Table 1:** Scale used for scoring adhesions present at necropsy in each group

	Adhesion scoring
0	No adhesions
1	Solitary adhesion to/from omentum; fibrinous and avascular; adhesion easily released with gentle digital traction
2	Omental adhesions or solitary adhesion to adjacent viscera or body wall; fibrinous/unorganized and avascular; adhesions easily released with gentle digital traction
3	Same as (2) but adhesions are organized, dense, and vascularized; required blunt dissection to free
4	Adhesions (omental, visceral, body wall); well organized dense, vascularized; required sharp dissection to separate
5	Extensive organized adhesions requiring sharp adhesiolysis

For histopathology, samples were taken from the antimesenteric site of the anastomosis, stained with haematoxylin and eosin, and examined by a blinded pathologist for inflammation and neovascularization. Sample slices were also stained with Masson’s trichrome to assess collagen content [5]. Histologic parameters scored on a scale from Hope et al. [38], included inflammation, collagen deposition and vascularity (Table 2).

**Table 2:** Scale used for histological evaluation of the anastomotic healing: value from 1 to 4 for the inflammation and 1 to 3 for vascularization and collagen content

Score	Inflammation: number of giant cells—(GC) and lymphocytes (L)	Collagen deposition (layers)	Blood vessels in mucosa at anastomosis
1	GC, L < 5	Thickness 1–3 layers	< 5
2	GC, L 5–10	Thickness 4–10 layers	6–10
3	GC, L 11–15	Thickness > 10 layers	> 10
4	GC, L > 15	–	–

### Statistical analysis

The distribution of data was evaluated using the Shapiro–Wilk test. We used the Repeated Measures ANOVA test for comparison of anastomosis times and bursting pressures (for normally distributed data), and a Friedman test to compare adhesions and histopathology scores (for data not normally distributed). All statistical analysis was performed using commercially available software^8^ with the significance set at P < 0.05.

## Results

All six pigs started eating 18 h after surgery and survived until euthanized. No postoperative complications were encountered. A total of 36 anastomoses (12 for each group [A, B, and C]) were performed for the study.

### Suturing time

Mean construction times were 518 ± 40 s for the A group, 487 ± 45 s for the B group, and 587 ± 63 s for the C group. Overall, anastomoses in the A group and B group were significantly faster to construct than in the C group (P = 0.0012). No difference was detected in construction time between the A and B groups.

### Necropsy findings

The omentum was adhered to the abdominal incision in four out of six animals. There was no evidence of stenosis, leakage or granuloma/abscess at the anastomotic sites. Adhesions between the anastomosis site and other portions of the small intestine, which were not involved in the anastomosis procedures were found in 3/12 anastomoses in the A group, 4/12 in the B group, and 3/12 in the C group. The median adhesion score was 1.5 in the A and B group, and 1 in the C group, but difference between these values was not significant (P = 0.81).

### Busting pressure

Bursting occurred at the mesenteric site in 12/12 intact intestinal samples and in 28/36 anastomoses (8/12 in group A, 10/12 in group B, and 10/12 in group C). Other sites where bursting occurred were the antimesenteric site (n = 4, two each in the A and C groups) and, in four cases, midway between the two (2/12 both in the A and B group). No suture and/or knot failure was detected, whereas tissue failure was a regular occurrence. Mean bursting pressures were significantly higher (P < 0.0001) in the intact intestine (198 ± 13 mmHg) than in any other group (group A, 150 ± 16 mmHg; group B, 145 ± 22 mmHg; group C, 145 ± 24). The bursting pressures were not significantly different between anastomotic techniques.

### Histology

No significant differences were found in any of the histological parameters (Table 3—median and range), but there was a non-statistically significant trend for lower values of neovascularization and inflammation in groups A and B (barbed suture), while collagen content was lower in group C (non-barbed suture).

**Table 3:** Histology results for the jejuno-jejunal anastomosis groups using different suture materials: total scores derived summarizing the values described in “Methods” section (1 to 4 for the inflammation, 1 to 3 for vascularization and collagen content)

	Collagen content	Neovascularization	Inflammation score
Group A	3.5 (2–4)	1 (1–3)	1.5 (1–2)
Group B	3 (2–4)	1 (1–3)	2 (1–3)
Group C	2 (1–4)	2 (1–3)	2.5 (1–3)
P-value	0.0583	0.0865	0.5719

## Discussion

Both tested barbed sutures proved to be safe and effective for one-layer, extramucosal, end-to-end, jejuno-jejunal anastomoses. In this regard, our results confirm experimentally the findings empirically reported in clinical settings.

Adverse effects mostly caused by exposed barbs, such as adhesions with other organs and intestinal obstruction, have been reported with the clinical use of barbed suture [27–30].

In our study, adhesions at the anastomotic site were encountered with both barbed suture materials although they did not occur in a significantly different percentage compared to unbarbed suture. This could be due to the suture pattern used or the fact that we tested these sutures in healthy animals. Using different suture patterns or operating in a clinical setting may lead to different results. Further, longer follow-up periods might have highlighted different complications.

Regarding both barbed sutures, extra care must be taken to position the needle accurately before each bite because the suture cannot be retrieved once in place [31]. Good tension control of each bite is essential for the same reason. An easy way to achieve this is by evenly applying tension on the stay sutures at the mesenteric site and the antimesenteric border of the anastomosis. In our case, two stay sutures were sufficient to avoid a purse-string effect, with no need for an additional suture as hypothesized in a previous study [31].

Overall, we found the main advantages with barbed sutures to be suturing time and handling, in accordance with previous studies [30]. In fact, since they are a knotless material, construction times were significantly shorter using the barbed sutures compared to the traditional suture. Even more advantageously, the barbs are specifically designed to self-engage into the tissue as the suture line proceeds. Not only did this result in a further reduction of surgical time, it also facilitated a more ergonomic suture technique, as it removed the need to apply tension on the suture while placing the following bites of the continuous pattern [4]. On the whole, the bidirectional device was easier to handle and appeared to provide less tissue drag, factors that may contribute to the reduction in surgical time recorded with this suture material. These characteristics are possibly due to its lower barb number and longer spacing between barbs compared to the unidirectional barbed suture [32] or owing to the different material (polydioxanone vs. glycomer 631).

Although statistically significant, reduction in surgical time in the laparotomy model studied here was minimal; but while this could be of little clinical relevance, we should not underestimate their usefulness, especially during difficult procedures. For these reasons, barbed sutures may be indicated in anastomoses performed in poorly accessible sites, or with extensive resections where time may be a determinant for a successful outcome.

Our study is not without limitations, the most obvious being related to the type of suture used as a control. We chose glycomer 631 because, out of all the options available, it is the most similar to the suture material used in group A, which has already been employed for gastrointestinal anastomosis. Other types of sutures might have caused a milder inflammatory response or yielded different results.

**Fig. 3:**
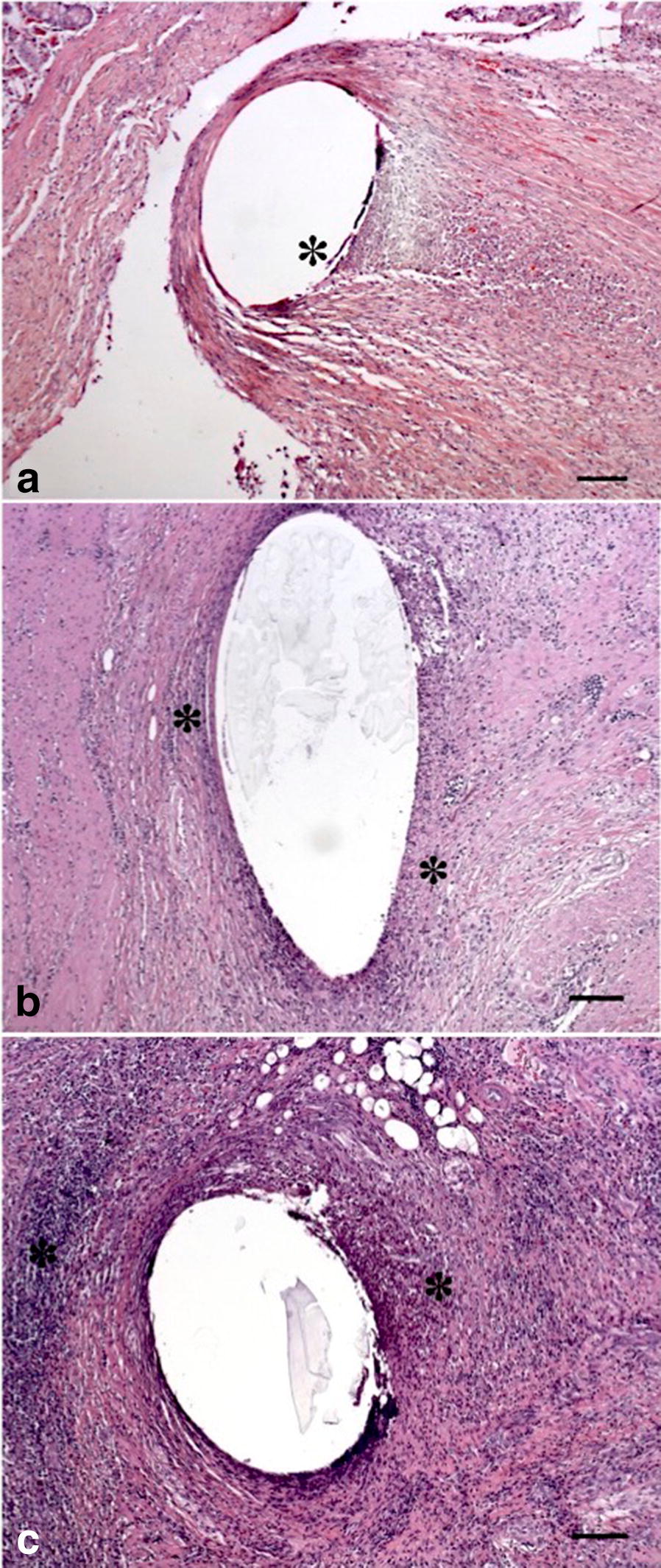
Photomicrograph of granulation tissue between submucosa and muscle layers at the anastomotic site for each group (**a** Byosin unbarbed suture, **b** unidirectional barbed suture, **c** bi-directional barbed suture). **a** Foreign material—suture surrounded by a large amount of inflammatory cells (lymphocytes and giant cells (*); **b**, **c** a large hole indicates the area of suture material, surrounded by a large number of inflammatory cells (*) and abundant fibrous tissue with a lot of collagen fibres. Hematoxylin–Eosin; Bar: 100 µm

Another limitation lies in the use of different suture materials within the same animal. Our selection aimed to avoid potential biases caused by individual reactions to the surgical procedure. This may, nevertheless, have taken a toll on the accuracy of the results and led to deceptively uniform inflammation scores (Fig. 3). Furthermore, this may not reflect the effective degree of inflammatory reaction to a given suture material.

As reported in previous work [1, 31], the choice of suture size had to take into account labeling differences. While unidirectional sutures are rated equal to traditional sutures in tensile strength, bidirectional sutures are rated one USP size smaller [1, 31]. This did not affect our findings, however, as knot or suture failure did not occur. Based on our experience, we recommend USP 4-0 as the smallest size of smooth, non-barbed suture employable for end-to-end jejuno-jejunostomies in pigs with an average weight of 35 kg. Finally, none of the suture materials cut through the tissues at any time during the procedures; nevertheless, sutures of varying sizes might have behaved differently.

In addition, we used an extramucosal appositional suture pattern for all procedures. A different pattern might have yielded different results, but, to date, no studies have compared the effects of suture pattern with barbed suture either in the intestine or in other tissues.

To the best of our knowledge, this is the first report on the in-vivo use of barbed suture materials for an end-to-end anastomosis in animals. Bidirectional barbed sutures proved just as effective as unidirectional barbed sutures and both were comparable to traditional, non-barbed sutures, but gave a statistically significant reduction in surgical time. This could pave the way to a wider use of barbed suture materials in open, as well as in laparoscopic, surgery. Unfortunately, barbed sutures are more expensive than smooth sutures of the same materials and this may limit their use in clinical practice.

## Conclusions

Both unidirectional and bidirectional barbed sutures can be safely and effectively used for appositional, extramucosal anastomosis in pigs. Barbed suture devices are comparable to non-barbed glycomer 631 in terms of anastomotic healing and suture-holding capacity, but barbed sutures are associated with reduced surgical time.

## Footnotes

1 Bayer Animal Health, Milano, Italy.

2 Virbac, Milano, Italy.

3 Covidien, Segrate, Italy.

4 Quill™ SRSSurgical Specialties Corporation, Wyomissing, PA, USA.

5 Fatro SpA, Bologna, Italy.

6 Schering-Plug, Milano, Italy.

7 MSD Animal Health srl, Milano, Italy.

8 GraphPad Software Inc, La Jolla, CA, USA.
